# Alizarin, an Agonist of AHR Receptor, Enhances CYP1A1 Enzyme Activity and Induces Transcriptional Changes in Hepatoma Cells

**DOI:** 10.3390/molecules28217373

**Published:** 2023-10-31

**Authors:** Shengxian Liang, Haimei Bo, Yue Zhang, Hongcheng Zhen, Li Zhong

**Affiliations:** 1 Institute of Life Sciences and Green Development, College of Life Sciences, Hebei University, Baoding 071000, China; haimei416@163.com (H.B.); izyue12h@163.com (Y.Z.); zhenhongcheng@126.com (H.Z.); 2 Department of Basic Medical Sciences, College of Osteopathic Medicine of the Pacific, Western University of Health Sciences, Pomona, CA 91766, USA

**Keywords:** alizarin, HepG2 cells, CYP1A1 activity, AHR pathway

## Abstract

The phytopigment alizarin was previously characterized as an anti-tumor drug owing to its antioxidant or antigenotoxic activities. However, the safety of alizarin is currently still under dispute. In this study, we explored the activity of alizarin in the AHR-CYP1A1 pathway and analyzed the transcriptional changes affected by alizarin using human hepatoma cell line HepG2-based assays. The results showed that alizarin decreased HepG2 cell viability in a dose-dependent manner, with IC_50_ values between 160.4 and 216.8 μM. Furthermore, alizarin significantly upregulated the expression of *CYP1A1* and increased the ethoxyresorufin-O-deethylase activity. Alizarin also exhibited agonistic activity toward the AHR receptor in the XRE-mediated luciferase reporter gene assay, which was further confirmed via the molecular docking assay. In addition, the transcriptional analysis indicated that alizarin may act as a potential carcinogen through significantly enriching several items related to cancer in both DO and KEGG analysis. In brief, our findings indicated that alizarin shows agonistic activities to the AHR receptor through activating the AHR-CYP1A1 signaling pathway in HepG2 cells, which may lead to the risks for cancer developing.

## 1. Introduction

Alizarin (1,2-dihydroxyanthraquinone) is a red coloring mordant dye, which was originally extracted from the roots of madder plants (*Rubia tinctorum* L). It has been used in textile and electronics industries and applied to determine the calcium deposits in cell cultures [1,2,3]. In addition, some studies proposed that alizarin may be used as an anti-tumor drug [4,5] with no mutagenic properties and antioxidant or antigenotoxic activities [6,7,8,9]. However, other researchers obtained opposite results that alizarin showed carcinogenesis-promoting properties [10,11,12]. Therefore, the roles of alizarin in tumorigenesis remain controversial.

Previously, alizarin was characterized as an inhibitor of the phase I metabolizing enzyme cytochrome P450-1A1 (CYP1A1) in engineered *E. coli* co-expressing recombinant human CYP1A1 and NADPH-cytochrome P450 reductase [7]. But assays with mice showed that alizarin exposure did not significantly affect the CYP1A1 enzyme activity in the liver [13]. The effects of alizarin on the human enzyme activity of CYP1A1 remains obscure. Studies have shown that the Aryl hydrocarbon receptor (AHR) mediates the induction of CYP1A1 [14] by binding to the sequence of xenobiotic-responsive elements (XREs) in the enhancer region of CYP1A1. The AHR-CYP1A1 pathway was associated with cancer progression [15,16,17] through metabolizing some xenobiotics into mutagenic epoxide intermediates or interacting with other signaling pathways that are responsible for cell invasion, migration, etc. Therefore, elucidating the effects of alizarin on the AHR-mediated CYP1A1 pathway with human cell-based assays may help us to better understand its carcinogenic or anticancer properties.

Human hepatoma cell line HepG2 has been extensively used as a model in assessing human health risks, as well as in investigating the effects of xenobiotics on the activation of AHR pathways. In this study, we explored the effects of alizarin on the AHR/CYP1A1 signaling pathway using HepG2 cells and investigated the transcriptional changes of HepG2 responding to alizarin exposure. We showed that alizarin is an agonist of the AHR receptor and increases the CYP1A1 activity in HepG2 cells, which may be partly through activating the AHR pathway.

## 2. Results and Discussions

### 2.1. Cytotoxicity of Alizarin with HepG2 Cells

**Figure 1:**
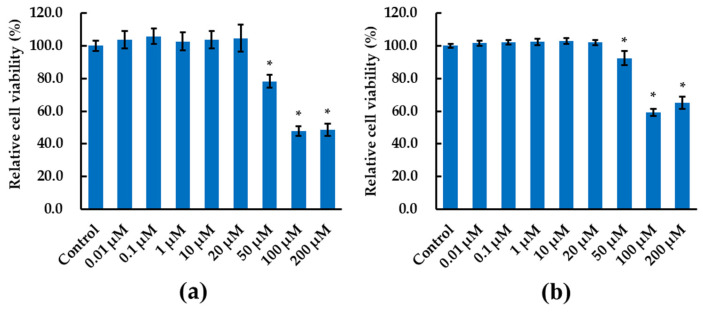
Alizarin exposure decreased the viability of HepG2 cells in a dose-dependent manner. HepG2 cells were exposed to various concentrations of alizarin bought from MCE (**a**) and TCI (**b**) for 48 h. The data are presented as mean ± SD, with at least nine replicates from two independent experiments. * *p* < 0.05 versus solvent control.

To avoid deviations induced by the heterogeneity of product quality, we assessed the cytotoxicity of alizarin that was bought from two different manufacturers. HepG2 cells were exposed to various concentrations of alizarin for 48 h, as shown in Figure 1, where alizarin did not affect the cell viability with concentrations lower than 50 μM, but it significantly impaired the cell viability at high concentrations (≥50 μM). Alizarin purchased from the two manufacturers showed a consistent trend, and the one bought from MCE showed more noticeable effects at higher concentrations (≥50 μM). The calculated IC_50_ value of alizarin in HepG2 cells was 160.4 μM (Figure 1a, MCE) and 216.8 μM (Figure 1b, TCI) in the present study, which are higher than the IC_50_ values in several pancreatic cancer cell lines SW1990 (22.1 μM), BxPC3 (35.9 μM), PANC-1 (15.6 μM), Ostesarcoma cell lines Sao-2 (27.5 μg/mL, 114.5 μM), MG-63 (29.0 μg/mL, 120.7 μM), and MIA PaCa-2 (10.2 μM) [4,5]. However, the IC_50_ values of alizarin in HepG2 are lower than the IC_50_ values in mesenchymal stromal cells (828.9 μg/mL, 3.5 M), breast carcinoma cell line MDA-MB-231 (62.1 μg/mL, 258.5 μM), Prostate carcinoma cell line PC-3 (241.3 μg/mL, 1.0 M), and U-2 OS (69.9 μg/mL, 291.0 μM) [5]. Moreover, alizarin also showed inhibitory function to the growth of freshwater microalgae (effective concentrations: 78.8–124.1 μM) and the biofilm formation of bacteria [18,19]. These results highlight the cell-dependent divergent effects of alizarin and its cytotoxicity to multiple organisms at high concentrations.

### 2.2. Alizarin Induced CYP1A1 Gene Expression and Increased Ethoxyresorufin-O-deethylase (EROD) Activity in HepG2 Cells

**Figure 2:**
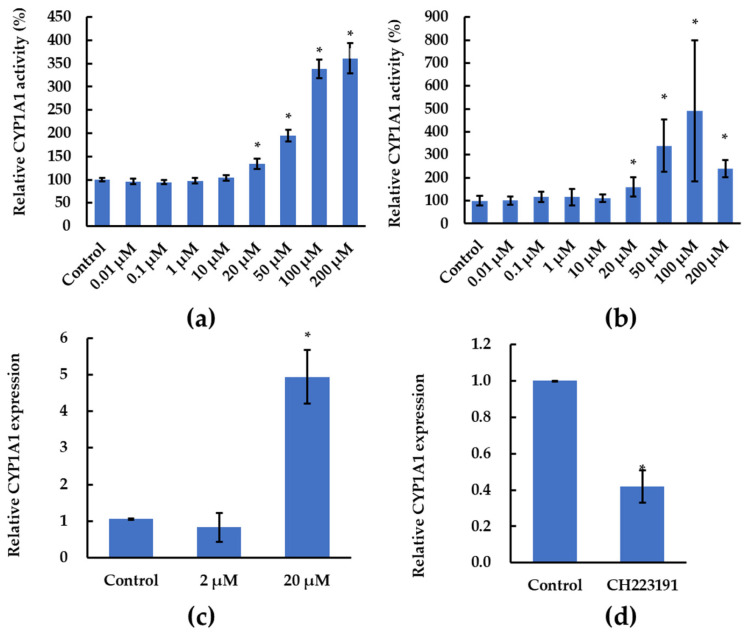
The activity of CYP1A1 enzyme in HepG2 cells that were exposed to alizarin for 48 h were determined via (**a**) EROD method and (**b**) Promega commercial kit, where the data are presented as mean ± SD, with 12 replicates in (**a**) and three replicates in (**b**). The relative mRNA expression levels of *CYP1A1* in HepG2 cells that were exposed to alizarin (**c**) and CH223191 (1 μM) (**d**) were determined, where the data are presented as mean ± SD of three independent experiments. * *p* < 0.05 versus solvent control.

We investigated the effects of alizarin on the activity of the CYP1A1 enzyme in HepG2 cells using two methods (the EROD assay and a commercial kit). As shown in Figure 2a,b, alizarin (≥20 μM) significantly increased the CYP1A1 enzyme activity in HepG2 cells, where comparable results were observed in HepG2 cells exposed to alizarin that was brought from another manufacturer (TCI, Figure S1). Moreover, we quantified the mRNA level of *CYP1A1* with the RT-qPCR assay, where results revealed that the expression level of *CYP1A1* was also upregulated by alizarin exposure (Figure 2c). The positive control showed that CH223191 (antagonist of AHR) significantly decreased the expression of *CYP1A1* (Figure 2d). These data suggested that alizarin is an activator to the CYP1A1 pathway. However, inconsistent with our results, previous studies characterized alizarin as an inhibitor of CYP1A1 in *E. coil* and as having no effects on the CYP1A1 enzyme in mice [7,13]. In Takahashi’s studies, they applied the engineering bacteria or mice to investigate the effects of alizarin on CYPs enzyme activities, which may not extrapolate directly to humans due to lacking the regulation networks of target enzymes in human cells [7,13].

Other anthraquinone derivatives including purpurin, 1-Hydroxyanthraquinone, and quinizarin that shared similar structures with alizarin could also modulate the cytochromes P450 enzyme activities [20]. Interestingly, contradictory effects of purpurin on CYP1A1 enzyme activity were observed in assays with engineered bacteria and cells or animals [7,13,20,21]. Therefore, as discussed above, more human relative testing assays should be performed to demonstrate the effects of these anthraquinone derivatives on the cytochromes P450 enzyme activities, which could exclude the inter-specific variations.

### 2.3. In Vitro and In Silico Study on the Interactions between Alizarin and AHR Receptor

**Figure 3:**
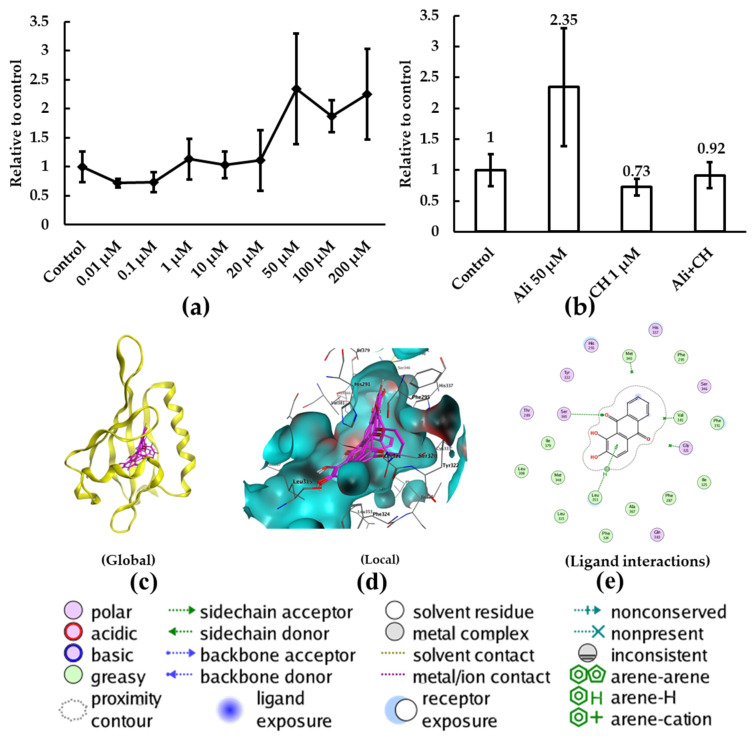
Analysis of the binding potential of alizarin to AHR_LBD. The activities of XRE-mediated luciferase of HepG2 cells were determined after treatment with alizarin or CH223191 (CH) (**a**,**b**), where the data were presented as mean ± SD of three replicates and the number above the volume in (**b**) represents the fold change compared to the control. The docking results of the interactions between alizarin and AHR_LBD were shown in (**c**) global view and (**d**) local view, the molecular surface of the protein was displayed according to the molecular properties, and red, cyan and black represents H-bonding, hydrophobic and mild polar, respectively. (**e**) 2D diagram interactions between alizarin and the AHR_LBD binding sites.

The nuclear receptor AHR is a ligand-dependent transcription factor that belongs to the basic helix–loop–helix PER-ARNT-SIM (bHLH-PAS) superfamily and plays critical roles in many physiological processes [22]. Numerous studies have shown that AHR directly regulates the increased expression of *CYP1A1*; therefore, we proposed that alizarin may regulate the *CYP1A1* expression and enzyme activity by interacting with AHR first. To explore the interactions between alizarin and the AHR receptor, we applied an AHR-mediated luciferase reporter gene assay. As shown in Figure 3, alizarin exposure significantly enhanced the transcriptional activity in a dose-dependent manner (Figure 3a), while the AHR antagonist CH223191 suppressed the effects of alizarin (Figure 3b), suggesting that alizarin was a potential agonist to AHR. Interestingly, previous studies found that alizarin could suppress the 2,3,7,8-Tetrachlorodibenzo-p-dioxin (TCDD)-induced AHR transformation in HepG2 or in cytosol containing AHR extracted from mammalian liver cells, suggesting an antagonistic activity towards the AHR receptor [23,24]. Though alizarin and TCDD have comparable binding abilities with AHR, they may have different effects in regulating the downstream transcription of target genes. In another word, when the more effective TCDD was partially replaced by alizarin, the expression of AHR target genes would be decreased compared to the TCDD treatment alone. Similar results were observed in MCF-7 cells, where the exposure of either galangin alone or TCDD alone increased the expression of *CYP1A1*, while galangin + TCDD co-exposure significantly decreased the expression of *CYP1A1* compared to the TCDD group alone [25]. In addition, other studies also suggested that the agonistic or antagonistic activities of phytochemicals may depend on the cell context and exposure concentrations [24,26].

To further characterize the binding interactions between alizarin and the human AHR ligand-binding domain (AHR_LBD), we employed molecular docking analysis. Since the AHR protein crystal structure was not available yet, we predicted the 3D structure of AHR-LBD with SWISS-MODEL. The best modeled human AHR_LBD is shown in Figure S2, with more than 99% amino acid residues clustered within the sterically favorable or allowable regions in the Ramachandran plot analysis, suggesting that the structure was relatively reliable. Consistent with previous study [27], the binding pocket of AHR was automatically searched and located at the center of the protein (Figure 3c) and occupied a mostly hydrophobic site (Figure 3e). Specifically, alizarin may form π-H with the side chain atoms of Val381 and Leu353, establishing hydrogen bond interactions with Gly321, Met340, and Ser365 (Figure 3e). Additionally, we applied TCDD as a positive control (Figure S3), where both alizarin and TCDD could form π-H with the side chain atom of Leu353 (Figure 3e and Figure S3). The average of the top five docking scores of alizarin and TCDD was −5.6 ± 0.2 and −4.9 ± 1.5 (Figure S4), respectively. These data further confirmed the binding potential of alizarin to AHR_LBD.

### 2.4. The Transcriptional Changes of HepG2 Cells Exposed to Alizarin

**Figure 4:**
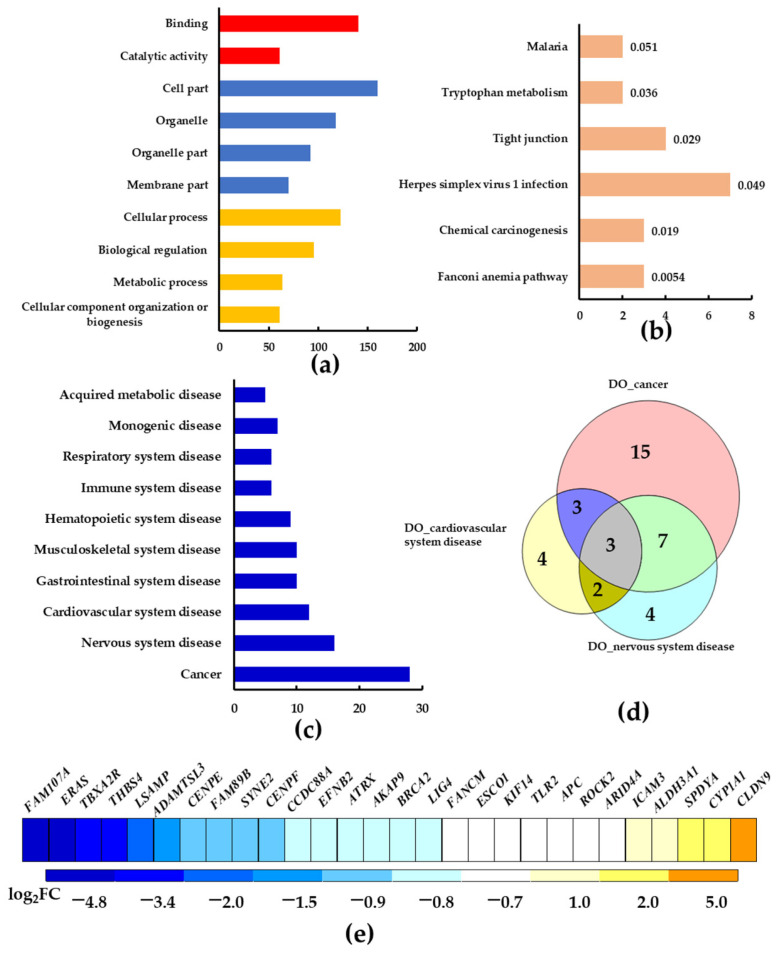
Analysis of the DEGs of HepG2 cells exposed to alizarin. (**a**) showed the top 10 GO annotation terms, and red, blue, and yellow represents the GO categories of “Molecular function”, “Cellular component”, and “Biological process”, respectively. (**b**) showed the significantly enriched KEGG pathways. (**c**) showed the top 10 DO annotation terms. (**d**) showed the Venn diagram analysis of the DEGs in the top three DO categories. (**e**) showed the 28 DEGs significantly enriched in the “Cancer” term in DO annotation analysis.

To obtain an overview of the effects of alizarin on HepG2 cells, we analyzed the whole transcriptional changes in HepG2 cells exposed to alizarin (20 μM) for 48 h. A total of 286 DEGs (differential expressed genes) were found in alizarin-treated samples, with 213 down-regulated and 73 up-regulated. GO (Gene Ontology) annotation analysis showed that these 286 DEGs were mostly relative to “cellular processes” and “biological regulation” in the GO category of “biological processes” (Figure 4a), suggesting that alizarin may damage some universal physiological processes of cells. Similarly, under the categories of “cellular component” and “molecular function”, some universal terms such as “binding”, “cell part”, and “organelle” were identified (Figure 4a). In KEGG (Kyoto Encyclopedia of Genes and Genomes) enrichment analysis, “Fanconi anemia (FA) pathway” was the most significantly enriched pathway, with down-regulated DEGs of *REV3L*, *BRCA2,* and *FANCM* (Figure 4b). Recent studies revealed that the FA pathway was involved in cancers and is critical in the maintenance of genomic stability [28]. Following the second significantly enriched pathway “Chemical carcinogenesis” (Figure 4b), it involves the DEGs of *KYAT1* (down-regulated), *CYP1A1* (up-regulated), and *ALDH3A1* (up-regulated). The KEGG data indicated that alizarin exposure may increase the risk of developing cancers.

To determine whether the DEGs in alizarin-treated cells were associated with human disease, the DO (Disease Ontology) analysis was performed. As shown in (Figure 4c), the term “cancer” contained the largest number of DEGs. Specifically, there are 28 DEGs in the category of “cancer”, with 23 down-regulated and five up-regulated, including *FANCM*, *BRCA2*, *KLF14*, *ERAS*, *FAM107A*, *ROCK2*, *CLDN9*, *CYP1A1*, *ALDH3A1*, etc. (Figure 4e). Additionally, the categories of “nervous system disease” and “cardiovascular system disease” contained 16 and 12 DEGs, respectively, which were also ranked in the top list. Moreover, the Venn diagram analysis revealed that the top three DO categories only shared three DEGs, while the “nervous system disease” and “cardiovascular system disease” shared 62.5% and 50% DEGs with “cancer”, respectively (Figure 4d). These results imply that alizarin may cause adverse effects relative to the nervous system and cardiomyocytes besides cancer; thus, it is necessary to assess the safety of alizarin with cells from other organs and tissues.

Though previous reports suggested alizarin could be used as an anticancer drug [4,5,29], up to now, no sufficient in vivo data were available to support this proposal. On the contrary, assays with rats indicated the carcinogenic potential of alizarin by showing greater distribution in the outer stripe of the outer medulla, increasing 8-OHdG levels and exerting tumor-promoting effects in the kidney [10,30,31]. Consistently, our data also indicated the carcinogenesis of alizarin, where the DO analysis for the DEGs induced by alizarin showed that “Cancer” was one of the most enriched categories (Figure 4c). Specifically, *FAM107A*, which was characterized as a putative tumor suppressor in pan-cancer analysis [32], was significantly down-regulated by alizarin (Figure 4e). In addition, as shown in Figure 2, alizarin significantly activated the AHR receptor and thus increased the expression of *CYP1A1*. As mentioned in the introduction part, the AHR-mediated CYP1A1 activation was associated with cancer progression [14,15,16,17]. These data suggested that alizarin exposure may increase cancer risks.

## 3. Materials and Methods

### 3.1. Chemicals and Cell Culture

Alizarin (CAS No. 72-48-0) was purchased from MedChemExpress (MCE) Company (Cat. HY-N0563, purity 99%, Monmouth Junction, NJ, USA) and TCI (Cat. D0242, purity > 95%, Tokyo, Japan). A total of 200 mM stock solutions were prepared with dimethyl sulfoxide (DMSO) and stored at −20 °C in the dark. Working solutions of alizarin were prepared with the cell culture media. The final concentration of DMSO was no more than 0.1%.

Human liver cancer cell line (HepG2) was purchased from the Cell Bank/Stem Cell Technology Platform, Shanghai Institute of Life Science, Chinese Academy of Sciences. HepG2 was cultured at 37 °C in humidified 5% CO_2_ incubators using DMEM media (Life Technologies, Waltham, USA) containing 10% fetal bovine serum (Life Technologies, Waltham, MA, USA, 12664025C), 1% penicillin/streptomycin (Sangon Biotech, Shanghai, China), and 1% non-essential amino acids (Life Technologies, Waltham, MA, USA).

### 3.2. Cell Viability Test

HepG2 cells were cultured in 96-well plates with an initial density of 10,000 cells per well. After 24 h, the cells were refreshed with media containing various concentrations of alizarin. Cell viability was determined after a 2-day culture with Cell Counting Kit-8 (CKK-8) (APE×BIO, Houston, TX, USA, K1018) following the manufacturer’s directions.

### 3.3. Total RNA Extraction and qRT-PCR Analysis

HepG2 cells exposed to alizarin for 2 days were collected with a Trizol universal reagent (TIANGEN, Beijing, China, DP424). Total RNA was extracted and reversely transcribed into cDNA with the FastKing RT Kit (TIANGEN, Beijing, China, KR116) following the manufacturer’s guidelines. The qRT-PCR test was conducted with CFX384™ or the CFX96™ Real-Time PCR detection system (Bio-Rad, Hercules, CA, USA). Primer sequences are listed in Table S1.

### 3.4. RNA-Seq

Total RNA was extracted as described above. The cDNA library and sequencing were carried out at Shanghai Majorbio Bio-pharm Technology Co., Ltd. (Shanghai, China) using Illumina NovaSeq 6000 platform. Data analyses were conducted using the online tools of Majorbio Bio-pharm Technology Co., Ltd. (Shanghai, China). Briefly, the reads were filtered and mapped to the reference genome (GRCh38.p13) with HISAT and aligned with Bowtie2. The gene expression level and the differential expression analysis were analyzed with RESM and DESeq2, respectively. KEGG and GO enrichment were analyzed with KOBAS and Blast2GO, respectively.

### 3.5. AHR Mediated Luciferase Reporter Gene Assay and EROD Activity Test

The activity of alizarin toward human AHR was assessed with the XRE-mediated luciferase reporter gene assay. Briefly, HepG2 cells were transfected with pGL4.75 [hRluc/CMV] and pGL4.43[luc2P/XRE/hygro] vectors following the guidelines of Lipofectamine3000 (Thermo Scientific, Waltham, MA, USA, L3000015). After 24 h, the cells were refreshed with media containing various concentrations of alizarin in a 96-well plate, with 20,000 cells per well. The luciferase activity was tested after a 24 h culture using a dual-luciferase reporter assay kit (Promega, Madison, WI, USA, E19103).

The enzyme activity of CYP1A1 was determined with the EROD assay [33,34]. Briefly, HepG2 cells were cultured and exposed to alizarin as described in the CKK-8 assay. After a 2-day incubation, the cells were refreshed with media containing 2 μM 7-ethoxyresorufin and then incubated at 37 °C with 5% CO_2_ for 30 min. The fluorescence was assessed using a microplate reader (Varioskan Flash, Thermo Scientific, Waltham, MA, USA) with an excitation wavelength of 544 nm and an emission wavelength of 595 nm.

### 3.6. Molecular Docking Analysis

A homology model of the AHR_LBD was performed as previously described [35]. Briefly, the protein sequence of AHR_LBD (accession number NP_001612.1) was obtained from the NCBI website (https://www.ncbi.nlm.nih.gov/, accessed on 30 March 2022), where a sequence of AHR was truncated before Pro275 and after Lys397. The 3D structure of AHR_LBD was constructed with SWISS-MODEL (https://swissmodel.expasy.org/, accessed on 31 March 2022), taking a murine transcriptional activator complex (PDB ID: 4f3l.1.B) as the template. The binding affinities of chemicals to AHR_LBD were determined using MOE software (version 2019) [35]. 2,3,7,8-Tetrachlorodibenzo-p-dioxin (TCDD, CAS 1746-01-6) was applied as a positive control.

### 3.7. Statistics

Two-tail Student’s *t*-test was applied to perform the statistical analysis assuming equal variance. *p* ≤ 0.05 was regarded as statistically significant unless otherwise specified.

## 4. Conclusions

In conclusion, with in vitro and in silico assays, our data demonstrated that alizarin increases the CYP1A1 activity and acts as an agonist to the AHR receptor in HepG2 cells. Moreover, AHR-CYP1A1 activation is associated with cancer progression and our transcriptional analysis also suggested that alizarin exposure may increase the risk of developing cancers; therefore, more in vivo and stringent tests should be performed for the safety of alizarin in the future.

## Data Availability

The data presented in this study are available in the article or in Supplementary Materials section here.

## Supplementary Materials

The following supporting information can be downloaded at: https://www.mdpi.com/article/10.3390/molecules28217373/s1, Figure S1: The activity of CYP1A1 enzyme in HepG2 cells that were exposed to alizarin (TCI); Figure S2: Ramachandran plot of the AHR model; Figure S3: Molecular docking analysis of TCDD; Figure S4: The docking scores of chemicals against AHR_LBD; Table S1: Primers used for qRT-PCR; Table S2: DEG comparison between qRT-PCR and RNA-seq data.
