# Supplementary material for: Alizarin, an Agonist of AHR Receptor, Enhances CYP1A1 Enzyme Activity and Induces Transcriptional Changes in Hepatoma Cells
Source: Molecules. 2023 Oct 31;28(21):7373. doi: 10.3390/molecules28217373 (PMC10650112; doi:10.3390/molecules28217373)
Supplement: Supplementary file 1 [file molecules-28-07373-s001.zip › molecules-2655014-supplementary.pdf]

molecules

MDPI

Article

Alizarin, an Agonist of AHR Receptor, Enhances CYP1A1 Enzyme Activity and Induces Transcriptional Changes in Hepatoma Cells

Shengxian Liang [1,*], Haimei Bo [1], Yue Zhang [1], Hongcheng Zhen [1] and Li Zhong [1,2,*]

[1] Institute of Life Sciences and Green Development, College of Life Sciences, Hebei University, Baoding 071000, China; haimei416@163.com (H.B.); izyue12h@163.com (Y.Z.); zhenhongcheng@126.com (H.Z.)

[2] Department of Basic Medical Sciences, College of Osteopathic Medicine of the Pacific, Western University of Health Sciences, Pomona, CA 91766, USA

* Correspondence: liangshengxiansr@hbu.edu.cn (S.L.); lzhong@westernu.edu (L.Z.)

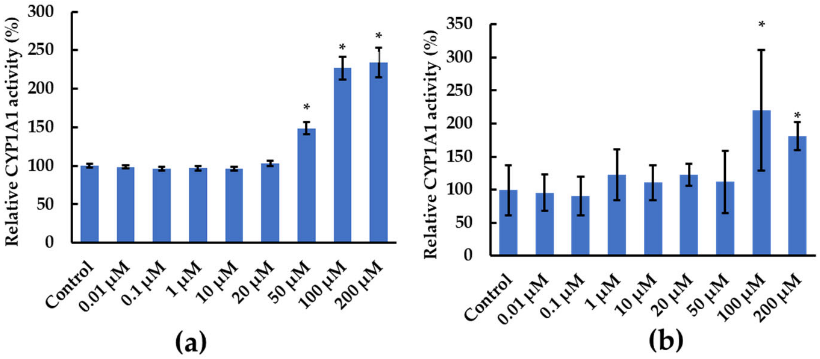

Figure S1. The activity of CYP1A1 enzyme in HepG2 cells that exposed to alizarin (TCI) for 48 h are determined by (a) EROD method and (b) Promega commercial kit, data are presented as mean ± SD, with 12 replicates in (a) and 3 replicates in (b). ∗ $p < 0.05$ versus solvent control.

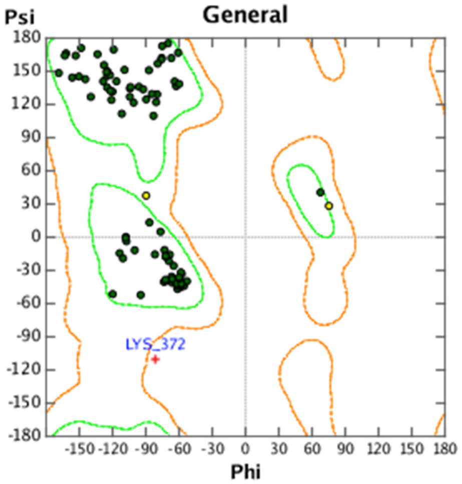

Figure S2. Ramachandran plot of the AhR model. Green dots represent steric favored, yellow dots represent steric allow, red dots reprensent steric repulsion.

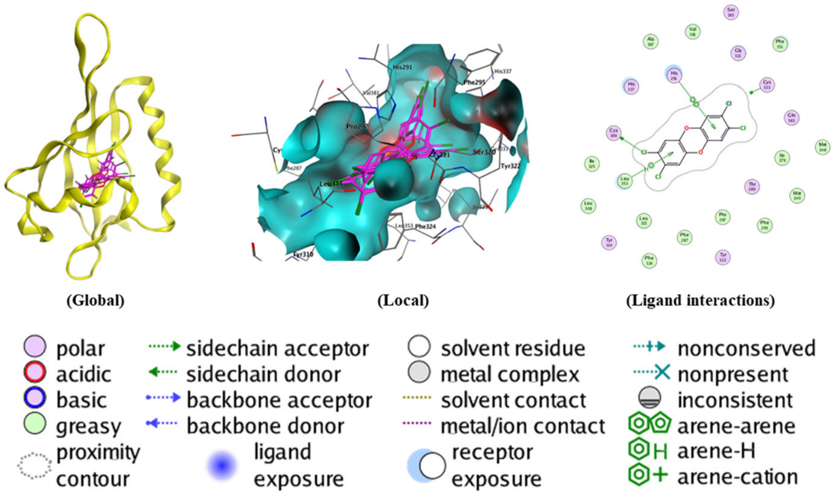

Figure S3. The docking results of the interactions between TCDD and AHR_LBD are shown in (a) global view and (b) local view, the molecular surface of the protein are displayed according to the molecular properties, red, cyan and black represents H-bonding, hydrophobic and mild polar, respectively. (c) 2D diagram interactions between alizarin and the AHR_LBD binding sites.

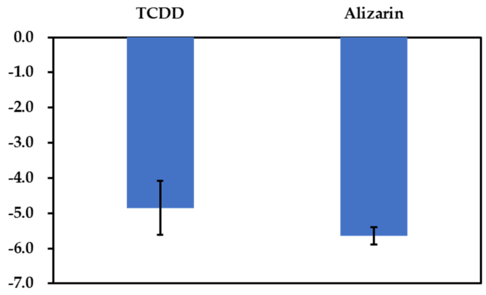

Figure S4. The average of the top five docking scores.

Table S1. Primers used for qRT-PCR.

Gene	Forward sequence	Reverse sequence	Product
GAPDH	TTGAGGTCAATGAAGGGGTC	GAAGGTGAAGGTCGGAGTCA	117 bp
ALB	TTGGCACAATGAAGTGGGTA	AAAGGCAATCAACACCAAGG	161 bp
CYP1A1	GAGGCCAGAAGAAACTCCGT	CCCAGCTCAGCTCAGTACCT	99 bp
UGT1A1	CCCATGCTGGGAAGATACTGT	GATGTACAACGAGGCGTCAG	131 bp
FABP1	GGAGGAATGTGAGCTGGAGACA	TATGTCGCCGTTGAGTTCGGTC	127 bp
AFP	ATTGGCAAAGCGAAGCTG	GCTGTGGCTGCCATTTTT	144 bp

Table S2. DEG comparison between qRT-PCR and RNA-seq data

Genes	qRT-PCR	RNA-seq
ALB	0.95	0.89
AFP	0.71	0.90
CYP1A1	4.94	4.67
UGT1A1	1.68	1.43
FABP1	0.87	0.91
